# The Use of Nanosecond Pulsed Fibre Laser Treatment to Improve the Corrosion Resistance of 316L SS Utilised as Surgical Devices

**DOI:** 10.3390/ma17246178

**Published:** 2024-12-18

**Authors:** Vinicius da Silva Neves, Felipe Queiroz Correa, Murilo Oliveira Alves Ferreira, Alessandro Roger Rodrigues, Witor Wolf, Rodrigo Galo, Fátima Maria Mitsue Yasuoka, Jéferson Aparecido Moreto

**Affiliations:** 1 Materials Engineering Department, São Carlos School of Engineering (EESC), University of São Paulo (USP), São Carlos 13563-120, SP, Brazil; neves.vineves@usp.br (V.d.S.N.); felipeqc@usp.br (F.Q.C.); moaferreira@usp.br (M.O.A.F.); witorw@gmail.com (W.W.); 2 São Carlos Institute of Physics (IFSC), University of São Paulo (USP), São Carlos 13566-590, SP, Brazil; fatimayasuoka@gmail.com; 3 Department of Mechanical Engineering, São Carlos School of Engineering (EESC), University of São Paulo (USP), São Carlos 13566-590, SP, Brazil; roger@sc.usp.br; 4 School of Dentistry of Ribeirão Preto, University of São Paulo (USP), Ribeirão Preto 14040-904, SP, Brazil; rogalo@forp.usp.br; 5 BR Labs Tecnologia Óptica e Fotônica Ltd., São Carlos 13567-020, SP, Brazil

**Keywords:** 316L stainless steel, laser treatment, corrosion, surgical instruments

## Abstract

The nanosecond pulsed fibre laser (NsPFL) treatment is extensively employed to distinguish hospital surgical instruments (micro-surgical forceps, surgical blades, orthopaedic drills, and high-precision laparoscopic tools), which are generally composed of stainless steel. Nevertheless, if the laser parameters are not properly optimised, this process may unintentionally provoke corrosion. Maintaining the structural integrity of these materials is essential for ensuring patient safety and minimising long-term costs. This work aims to optimise the laser scanning parameters for marking 316L stainless steel (316L SS), seeking to improve its corrosion resistance. The corrosion behaviour was assessed by using open circuit potential (OCP), potentiodynamic polarisation curves (PPc), and electrochemical impedance spectroscopy (EIS) techniques, conducted in 0.9% wt NaCl solution at a controlled temperature of 25 ± 1 °C. A comprehensive study employing optical profilometry has significantly enhanced our understanding of the corrosion micromechanisms of 316L SS, comparing specimens both with and without NsPFL treatment. Considering applications involving environments rich in chloride ions, the results indicated that the NsPFL-316L SS samples demonstrated markedly enhanced performance compared to the untreated base material after 48 h of immersion in 0.9% wt NaCl solution. This improvement is particularly noteworthy given the widespread utilisation of 316L SS in the manufacturing of surgical instruments, where corrosion resistance is of paramount importance.

## 1. Introduction

**Figure 1:**
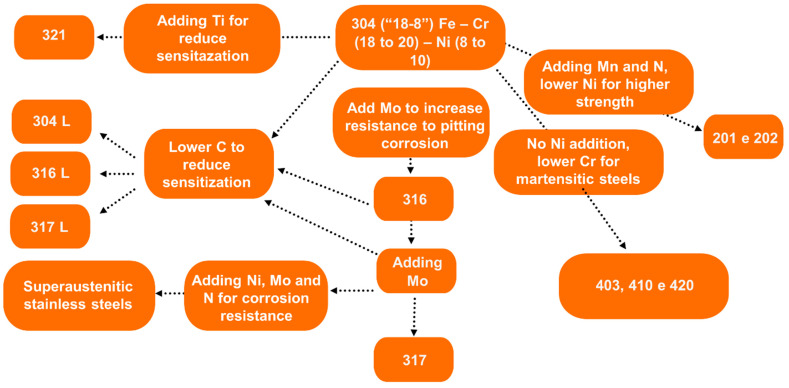
Schematic representation of the chemical composition adjustments required to produce 316L SS.

Numerous industrial sectors, including chemical, petrochemical, biomedical and aerospace, actively seek materials that combine exceptional mechanical properties with superior corrosion resistance. Once in these sectors, they often operate in challenging environments where exposure to aggressive chemicals, high temperatures, and mechanical stress is common. Consequently, the demand for advanced materials that not only withstand such harsh conditions but also maintain structural integrity over time is critical. In this context, materials that offer both mechanical and corrosion resistance are essential for enhancing the durability and longevity of equipment, components, and structures within these sectors. As a result, research and development efforts are increasingly focused on the exploration and optimisation of materials that can fulfil these stringent requirements, thereby driving innovation and efficiency in these vital sectors. Stainless steels, first developed in the early 20th century, have emerged as versatile materials with a wide range of applications [1,2,3]. Among the various types of stainless steels available on the market, 316L stainless steel (316L SS) has garnered significant attention in recent years, particularly in the biomedical field, due to its excellent biocompatibility and lower cost compared to conventional titanium-based alloys [4,5]. This steel is derived from 304 stainless steel (304 SS), commonly referred to as “18-8” stainless steel, which is characterised by a composition of 18–20 wt% Chromium (Cr) and 8–10 wt% Nickel (Ni). The development of 316L SS involves composition modifications to enhance its properties: increasing Ni content to 10–14 wt% to stabilise the austenitic phase, reducing Cr content to 16–18 wt%, and incorporating 2–3 wt% Molybdenum (Mo) to improve corrosion resistance. Furthermore, the designation “L” in 316L SS refers to its reduced Carbon (C) content, specifically designed to mitigate sensitisation processes [6]. Figure 1 shows a schematic representation of the chemical composition adjustments required to produce 316L SS.

The corrosion resistance of 316L SS is primarily attributed to the formation of a chromium oxide (Cr_2_O_3_) layer when exposed to the atmosphere [4,7,8]. This passive layer acts as a barrier, protecting the metallic matrix from environmental effects. However, under more aggressive conditions, such as the presence of chloride ions or environments with fluctuating pH levels, typical of the human body, 316L SS is susceptible to localised corrosion processes [9]. Several nanoengineering solutions have been developed to enhance the surface properties of various materials across a wide range of applications. These include anodising processes [10,11], nitriding processes [12,13], sol-gel coatings [14,15], polymer-based films [16,17], physical vapour deposition (PVD) and plasma-enhanced chemical vapor deposition (PECVD) [18,19,20,21,22,23], and laser treatments. The nanosecond pulsed fibre laser (NsPFL) technique is an advanced method used in various applications, including materials processing, surface modification, and laser machining [24,25,26]. The NsPFL involves the emission of laser light in short pulses, typically lasting in the nanosecond range, which allows for precise control over the laser’s energy delivery and interaction with the material [27,28]. Although the utilisation of NsPFL may lead to the degradation of the Cr_2_O_3_ passive layer formed on the 316L SS surfaces, this adverse effect can be mitigated through the careful regulation and optimal combination of laser parameters, including power, speed, and frequency, among others [29,30]. In this sense, this process not only modifies the microstructure of the material’s surface but also facilitates the formation of a more robust and durable passive layer, which serves as a barrier against corrosive agents present in the environment.

This study focuses on refining nanosecond laser scanning parameters to achieve precise and durable markings while maintaining surface integrity. The goal is to identify conditions that simultaneously allow effective marking and enhance corrosion resistance, ensuring the longevity and safety of 316L SS used as surgical devices.

## 2. Materials and Methods

**Figure 2:**
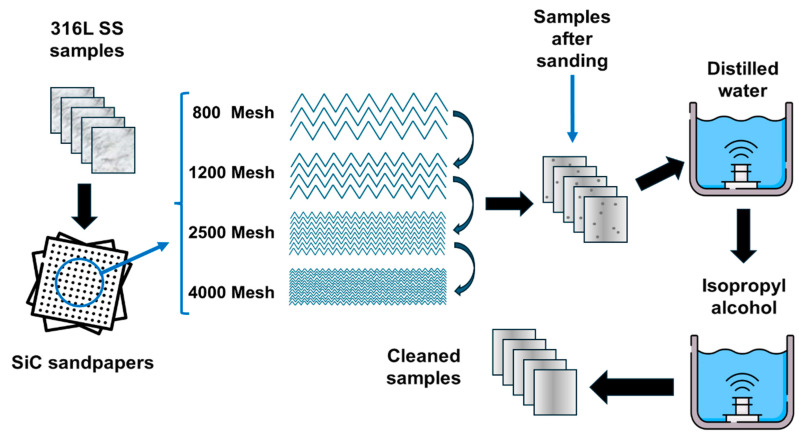
Schematic representation of the polishing steps of 316L SS prior to the NsPFL treatment.

Before the surface modification of 316L SS via laser, samples with dimensions of 15 × 15 × 2 mm^3^ were cut using electroerosion and subjected to a sanding process with silicon carbide (SiC) abrasive papers in the sequence range 800, 1200, 2500, and 4000 mesh. Final polishing was performed using 3 μm diamond paste. Subsequently, the samples were rinsed in distilled water and isopropyl alcohol for 15 min each and were placed in suitable sample holders. Figure 2 shows a schematic representation of the polishing steps of 316L SS prior to the laser treatment process.

**Figure 3:**
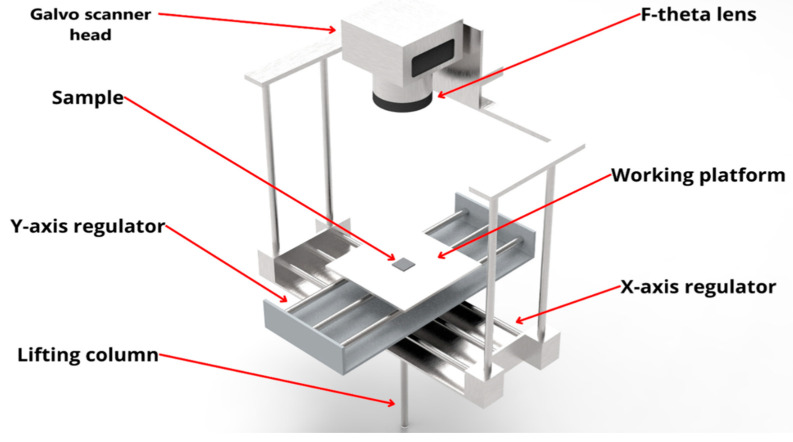
Schematic illustration of the NsPFL setup used on markings.

The surface treatment and texturization process was conducted by using NsPFL (SPI Pulsed Fiber Laser 70 W EPZ) with a power output of 70 W and a wavelength of 1060 nm, equipped with a two-axis galvanometer scan head. The 70 W SPI Pulsed Fiber Laser system, operating at a wavelength of 1060 nm, combined with a two-axis galvanometer scan and an f-theta lens that achieves a focal spot size of 18 µm, is a highly efficient and precise setup for industrial marking applications. The 18 µm focal spot size provides a concentrated high-energy density beam that allows for intricate detailing and fine control in texturization processes. In the marking system assembled by BR Labs, key marking parameters—such as power, scanning speed, number of loops, line spacing, and frequency—are configured using the EzCad software (14.13 version) (Laser marking and engraving software). This software provides a user-friendly interface to control and optimise the laser’s performance for different applications [31,32]. The marking parameters were meticulously optimised based on prior studies conducted by the research group: a power of 7 W, a scanning speed of 450 mm s^−1^, line spacing of 30 μm, frequency of 50 kHz, and a positioning adjustment on the z-axis precisely at the laser’s focus in 183 mm. Figure 3 exhibits a schematic representation of NsPFL setup used for marking applications. The galvanometer scan head allows rapid and precise movement of the laser beam across the surface in the x and y axes.

Uniform corrosion tests were performed by using a μStat-i 400s/MetrOhm potentiostat/galvanostat and an electrochemical cell containing a three-electrode compartment, consisting of a working electrode with an exposed area of 1 cm^2^ (samples of 316L SS with and without laser treatment), a platinum (Pt) counter electrode, and a saturated calomel reference electrode (SCE) (Hg/Hg_2_Cl_2_, KCl_sat_). The open circuit potential (E_ocp_) was monitored for a period of 10,800 s. Potentiodynamic polarisation curves (PPc) were obtained within a potential range of −0.3 to +1.2 V/SCE, with a scan rate of dE/dt = 0.1 mV s^−1^. The EIS spectra were recorded over a frequency range of 100 kHz to 10 mHz, employing a sinusoidal wave of 10 mV (rms). All the electrochemical tests were conducted in a 0.9 wt% NaCl solution, which was naturally aerated and maintained at a temperature of 25 ± 1 °C. For EIS data fitting, the *ZView2* software was used. Corrosion tests were conducted at the Department of Materials Engineering (SMM) of the São Carlos School of Engineering (EESC) at the University of São Paulo (USP), located in São Carlos, São Paulo, Brazil. The surfaces of both treated and untreated specimens were analysed by profilometry (Optical Profilometer Veeco model Vyko NT1100 embedded with Vision Software version 4.20) before and after corrosion testing at the Department of Mechanical Engineering of the São Carlos School of Engineering (EESC) at the University of São Paulo (USP), São Carlos, São Paulo, Brazil. Scanning Electron Microscopy (SEM) was also used to evaluate the treated and untreated specimen’s surfaces, after corrosion testing. The micrographs were taken using a secondary electron (SE) detector. For this purpose, a FEI Inspect S50 SEM microscope with a Tungsten electron gun, operating at 20 kV, was used in the Materials Engineering Department (DEMA) at the Federal University of São Carlos (UFSCar), São Carlos, São Paulo, Brazil.

## 3. Results and Discussion

**Figure 4:**
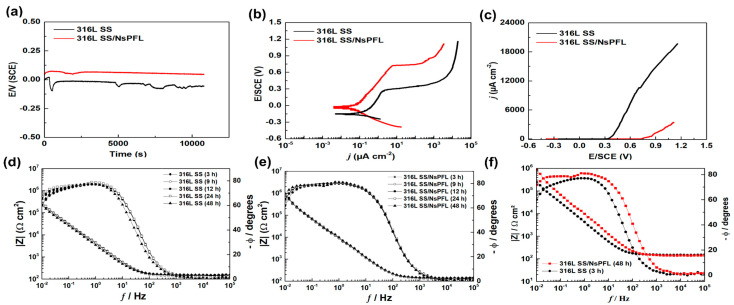
(**a**) Open circuit potential variation with time for 316L SS untreated and treated with NsPFL in aerated 0.6 mol L^−1^ NaCl solution, (**b**) potentiodynamic polarisation curves, (**c**) j versus E/SCE curves for determination of the pitting potential (E_pitting_), and (**d**–**f**) Bode plots.

Figure 4a presents the representative curves of the open circuit potential (E_ocp_) evolution as a function of immersion time for 316L SS, with and without NsPFL treatment. The results indicate that the E_ocp_ of the treated material is less negative (48 versus −58 mV/ECS), which may suggest a greater resistance to the corrosion process in a medium containing chloride ions. The characteristic polarisation curves (PPc) obtained for 316L SS with and without treatment are shown in Figure 4b. As observed, the treated material exhibited a corrosion potential (E_i=0_) that is slightly more positive than that of the base material. The value of E_corr_ is generally considered an indicator of the material’s nobility, with a higher E_corr_ corresponding to a system that is more resistant to corrosion [20]. The results of the PPc further demonstrated that the treated material has a lower corrosion current density (i_corr_) compared to the base material (0.0378 versus 0.1137 μA cm^−2^), confirming the effectiveness of the NsPFL treatment. The pitting potential (E_pitting_) for the base material is approximately 0.35 V/SCE compared to 0.75 V/SCE for the treated material (see Figure 4c).

The impedance spectra were obtained for at least three samples of each material studied: 316L SS and 316L SS treated with NsPFL, after 48 h of exposure to a 0.9% wt NaCl solution. In this paper, the immersion times of 3 h, 9 h, 12 h, 24 h and 48 h are presented, as illustrated in the Bode plots in Figure 4d,e, where the immersion times of 3 h and 48 h are compared (Figure 4f). An overview of the EIS spectra reveals the presence of two-time constants: one at high frequencies and another at low frequencies. The time constant observed at high frequencies is associated with the coatings produced by NsPFL on the 316L SS surfaces, whilst the time constant at low frequencies is directly related to localised corrosion processes. Indeed, the Bode plots (Figure 4d–f) reveal a substantial increase in the impedance moduli of the treated material, which intensifies with immersion duration and is accompanied by a phase angle nearing −90°. This behaviour indicates an enhanced resistance to the corrosion process in comparison to untreated 316L SS. These results highlight the beneficial effect of NsPFL treatment on the corrosion resistance of 316L SS.

**Figure 5:**
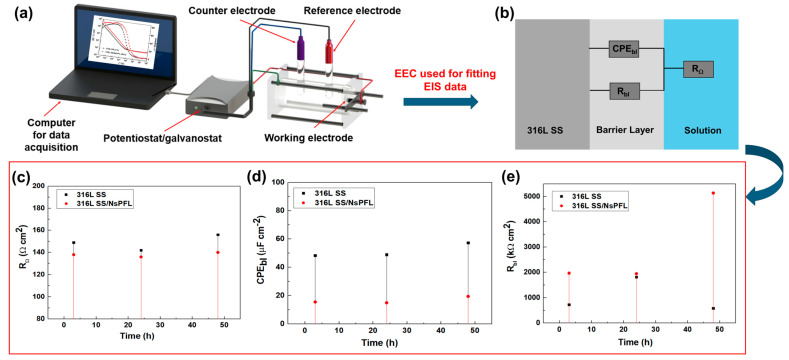
(**a**) Setup used on the corrosion tests, (**b**) EEC used for fitting the EIS experimental data, (**c**) R_Ω_ versus time, (**d**) CPE_bl_ versus time, and (**e**) R_bl_ versus time behaviour.

Figure 5a presents the electrical equivalent circuit (EEC) employed to elucidate the corrosion behaviour of 316L SS treated and untreated with NsPFL, alongside the electrochemical parameters obtained throughout the EIS data fitting procedure (see Figure 5b–d). The EEC comprises the solution resistance (R_Ω_), the polarisation resistance of the barrier layer (R_bl_), and the constant phase element of the compact barrier layer (CPE_bl_) [33]. The impedance of a CPE is given by the following equation:

(1) $$Z_{CPE}=\frac{1}{T{\left(j\omega \right)}^{\phi }}$$

where *T* is a parameter related to capacitance and ϕ is the constant phase exponent, with 0<ϕ<1.

As mentioned in reference [20], ϕ values become lower than 1 for the heterogeneous surfaces, and the *T* value does not represent the capacitance, despite having very close values. To facilitate the EIS interpretation, in the present paper the CPE_bl_ parameter will be considered as capacitance (C_bl_). As can be seen in Figure 5b, the typical values of the fitting results parameters for 316L SS and 316L SS/NsPFL indicated that the R_Ω_ remains constant across all immersion times, ensuring that the distance between the WE and RE was maintained consistently throughout the corrosion tests [20]. Regardless of the immersion time analysed, the values of the CPE_bl_ parameter were always higher for the 316L SS when compared to 316L SS/NsPFL as displayed in Figure 5c. Given that the C_bl_ can be represented as the following equation:

(2) $$C_{bl}=C_{bl}^{*}\left(\frac{A_{pitting}}{A_{geom}}\right)$$

where A_pitting_ represents the area that suffered pitting corrosion and A_geom_ represents the geometric area, it can be asserted that the increase in C_bl_ values for 316L SS is linked to heightened localised corrosion activity. As expected, the opposite effect was observed for the R_bl_ parameter as demonstrated in Figure 5d, corroborating the other results presented. ϕ_bl_ values were obtained between 0.87 and 0.95 for both studied materials. Ultimately, the results presented in this study enable us to assert that the treatment of 316L SS via NsPFL significantly enhanced its corrosion resistance in environments containing chloride ions.

**Figure 6:**
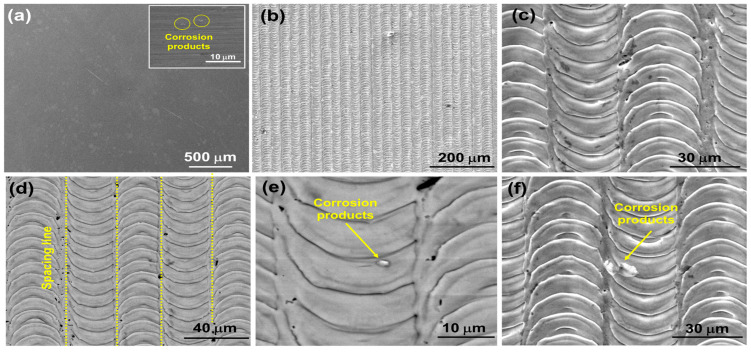
SEM images of the (**a**) 316L SS specimen, and (**b**–**f**) 316L SS/NsPFL specimen following EIS testing in 0.9 wt% NaCl solution. Images were obtained using a Secondary Electron Detector at different magnifications.

**Figure 7:**
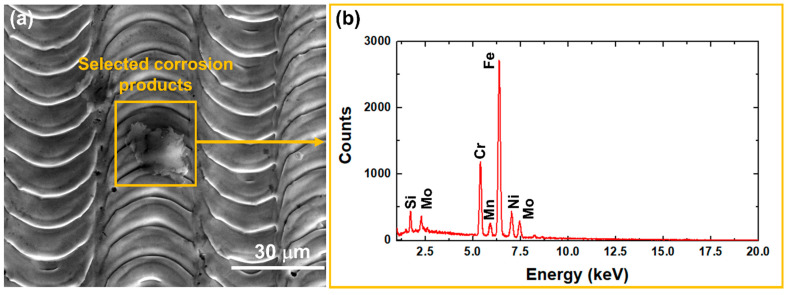
(**a**) SEM image, showing the selected corrosion products, and (**b**) its respective EDX spectrum, displaying the presence of Si, Mo, Cr, Mn, Fe, and Ni elements.

**Figure 8:**
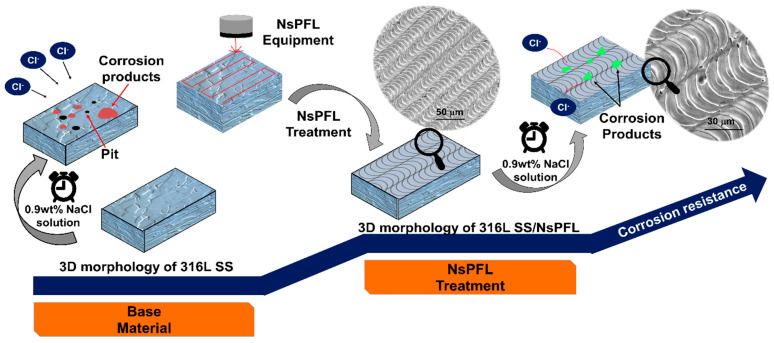
Schematic representation of the corrosion mechanism associated with the 316L SS under an aggressive environment containing chloride ions.

The enhanced corrosion behaviour of 316L SS/NsPFL can be attributed to the formation of a surface layer of corrosion products, which partially occludes the electroactive regions. Figure 6 displays the SEM micrograph images for the 316L SS and 316L SS/NsPFL samples after the EIS tests in 0.9 wt% NaCl solution. Figure 6a shows the surface of the 316L SS following the EIS corrosion test. In the upper right corner, a detailed view highlights the corrosion products that were observed distributed across the metallic surface. Figure 6b–f further illustrates the textured surface of the 316L SS at various magnifications, revealing the morphological characteristics and the extent of surface alterations induced by the corrosion process. As observed in Figure 6e,f, the corrosion products exhibit varying distributions within the matrix of the 316L SS treated by NsPFL. These corrosion products can be identified in the surface texture’s elevated peaks and recessed valleys. This distinct distribution not only underscores the heterogeneous nature of the corrosion process but also suggests significant interactions between the corrosion mechanisms and the surface topography of the treated 316L SS. Figure 7 presents the EDX spectrum of one of the corrosion products found on the 316L SS/NsPFL surface post-EIS testing. The primary elements identified in the analysed area, which could be quantified, are iron, chromium, and nickel. These findings suggest that the chemical composition of the corrosion product closely resembles that of the 316L SS matrix. It is important to note that the EDX technique did not allow for differentiation between the oxygen content in the metallic matrix and that within the corrosion product, implying the formation of oxides of a similar nature. Marjetka Conradi et al. [34], studied the localised corrosion process of 316L SS and duplex 2205 SS in simulated body solutions, and demonstrated, by using an X-ray Photoelectron Spectroscopy (XPS) technique, that the nature of corrosion products formed on the metallic matrix is composed of a combination of high state oxides, such as CrO_3_, NiO_2_ or Fe_3_O_4_. Figure 8 provides a schematic representation of the corrosion mechanisms related to 316L SS and 316L SS/NsPFL specimens when subjected to an aggressive environment rich in chloride ions. This illustration elucidates the various stages of the corrosion process, emphasising the occurrence of pits and the corrosion products on the base material surface. On the other hand, laser treatment possesses the capability to significantly enhance the corrosion resistance of 316L SS.

Vamsi Krishna Balla et al. [35] investigated the effects of laser surface melting (LSM) employing a continuous-wave Nd-YAG laser in an argon atmosphere at 1 and 5 mm s^−1^ on medical-grade 316L SS, and demonstrated that the corrosion protection efficiency of 316L SS was improved up to 70% by LSM in its as-processed condition. In reference [36], the potentiodynamic polarisation measurements were performed in the range −0.25 V versus E_OCP_ to  +  1.6 V by using Hank solution at room temperature. The results from corrosion testing conducted on both LSM and untreated samples in Hanks’ balanced salt solution indicated a significant reduction in i_corr_ values for the laser-treated 316L SS. Specifically, the i_corr_ was reduced to 0.729 μA cm^−2^ (1 mm s^−1^) and 0.237 μA cm^−2^ (5 mm s^−1^), compared to 2.465 μA cm^−2^ for the untreated 316L SS. Here, the i_corr_ value for the material treated with NsPFL is 0.0378 μA cm^−2^, despite the use of a 0.9 wt% NaCl solution. Another study that complements the current work was conducted by Shaahin Mohammadzadeh Asl et al. [36], in which nHA-PLA composites were applied to 316L SS via dip-coating, followed by using continuous mode CO_2_ laser treatment in the scan speed range of 1–10 mm s^−1^. The electrochemical study conducted in simulated body fluid (SBF) at 37 °C demonstrated a significant enhancement in the corrosion resistance of 316L SS, resulting from the laser treatment of nHA-PLA composite coatings. As reported by the authors [36], the improvement in the corrosion behaviour of 316L SS may be attributed to the defect-free, smooth surface with densified microstructure caused by CO_2_ laser treatment. In addition, authors mentioned that the reduced transport of electrons and ions between the 316L SS and SBF solution, facilitated by the laser-treated hydroxyapatite (HA) barrier coating, led to a reduction in both the electrochemical reactions and the corrosion rate. Regarding i_corr_ values, CO_2_ laser treatment was able to improve the corrosion resistance: 316L SS (8.91 μA cm^−2^), 316L SS+HA 10 min (5.82 91 μA cm^−2^), 316L SS+HA 2 min + laser (4.36 μA cm^−2^), 316L SS+HA 5 min + laser (3.98 μA cm^−2^), and 316L SS+HA 10 min + laser (3.80 μA cm^−2^).

Further analyses using profilometry could provide valuable insights into the depth and extent of these features, allowing for a more comprehensive discussion of the material’s degradation. Confocal profilometry assessments can help quantify numerous parameters and correlate them with the observed corrosion patterns, enhancing our understanding of how surface morphology influences corrosion resistance and material performance. The next paragraphs will be dedicated to this discussion to better understand the corrosion mechanism process of 316L SS with and without NsPFL treatment.

Table 1 shows the topographical parameters applied to characterise the specimens’ surfaces before and after the corrosion tests. A significant alteration in the surface morphology of the samples was identified due to the laser beam incidence. The sanded sample presented a roughness of 6.25 nm Sa (average) and 52.64 nm St (peak-to-valley) demonstrating that the sanding process was carefully carried out since the roughness was equivalent to those obtained in diamond turning machining [37]. Such a surface presents symmetry of the asperity heights about the mean plane (skewness) almost as Gaussian with peak prevalence (Ssk = 0.03 > 0), and the nature of the heights distribution (kurtosis) also near Gaussian curve with bumpy profiles varying gradually over the surface (Sku = 2.95 < 3.0) totalising almost 5000 summits per area (Sds = 4993.95 mm^−2^). Although the sanding process produced nanometric roughness, the summit density was relevant when compared with samples subjected to NsPFL and corrosion tests (discussed further). It is worth mentioning that summits are quite different from peaks, i.e., summits are found only above a threshold that is 5% of Sz roughness above the mean plane, and peak is defined as any point that is above all eight nearest neighbours separated by at least 1% of the minimum X and Y dimension comprising the 3D measurement area. Thus, besides peaks the summits are also considered in this study because they can help to explain the mechanism of improved corrosion properties promoted by NsPFL in conjunction with image analysis such as profilometry as well as SEM.

A very high spatial directionality or texture anisotropy was found on the sanded surface (Str = 0.07) since the texture aspect ratio tended toward zero. Thus, the sanding process produced a roughness in the dominant direction. The surface index (SI) of the sanded sample is only around 0.0030%, which means that the real sanded area is greater than its projected area (non-planned) of this percentile value. Finally, the volume (V) comprised between the highest peak and the lowest valley per analysed area unit was found to be 0.01803 μm^3^/μm^2^ [38]. The NsPFL-treated samples presented distinct roughness parameters when compared with the sanded one. Texturing the mechanism via heat input and the micrometric displacement of melted material increased the average (Sa) and maximum (St) roughness to 746.02 nm (+119 times) and 4020 nm (+76 times), respectively. Nonetheless, the predominance of peaks (Ssk = 0.08 > 0) and topography without inordinately spiked high peaks or deep valleys (Sku = 1.74 < 3.0) retained the same behaviour as the symmetry and distribution of the surface heights regarding the sanded specimen. Summit density (Sds = 2065.54 mm^−2^) was reduced by 58.64% because the laser beam melts the surface material and smooths the peaks, but the laser texturing by raster strategy (parallel engraving) also maintained the high spatial anisotropy (Str = 0.03~0). The major differences were found in the surface mean volume parameters, since the surface index (SI) and normalised volume (V) reached 5.2093%, and 1.47097 μm^−3^/μm^−2^, respectively. When compared with the sanding process, the increments in terms of area and volume to retain debris or house-deformed material from the laser sample surface were 1736.4 times, and 81.5 times, respectively.

Once the sanded and NsPFL-texturized surface characterisations (reference surfaces) have been discussed, their comparison with those under corrosion tests can be carried out. After the corrosion tests of the sanded sample, the average (Sa) and maximum roughness (St) are kept constant for both PP and EIS techniques when considering the results error range. The same behaviour took place in the NsPFL-texturized sample except for the PP method, which decreased by 24.26%. However, the sanded surface after the corrosion test changed the roughness height symmetry and distribution pattern related to the sanded sample. Although still near the Gaussian profile, the tested sample presented valley dominance (Ssk = −0.46 and −0.49 < 0) with inordinately spiked high peaks or deep valleys (Sku = 3.34 and 3.15 > 3.0). A similar mechanism occurred for NsPFL-treated surfaces for both EIS and PP techniques in terms of skewness (−0.45 and 0.03 < 0), but Kurtosis maintained bumpy profiles varying gradually on the surface (2.65 and 2.31 < 3.0). These behaviour regarding the roughness symmetry and distribution after corrosion tests demonstrate the dependence on the initial topography, but not on the corrosion testing method. For the initial finished roughness, the corrosion tests sharpen isolated peaks and valleys on the topography whereas peaks and valleys are rounded when the initial surface is rougher. On the one hand, this result relates directly to the summit density (Sds) because spiky peaks or valleys from the sanded sample diminished the amount of ridges for 1403.11 mm^−2^ and 1286.21 mm^−2^, respectively, after EIS (−71.90%) and PP (−74.24%) corrosion tests. On the other hand, when given the rougher NsPFL-texturized surface, the corrosion test increased the summit density over bumpy and uniformly spaced peaks or valleys by reaching 3172.60 mm^−2^ and 2518.72 mm^−2^ for EIS (+53.60%) and PP (+21.94%) techniques, respectively.

**Figure 9:**
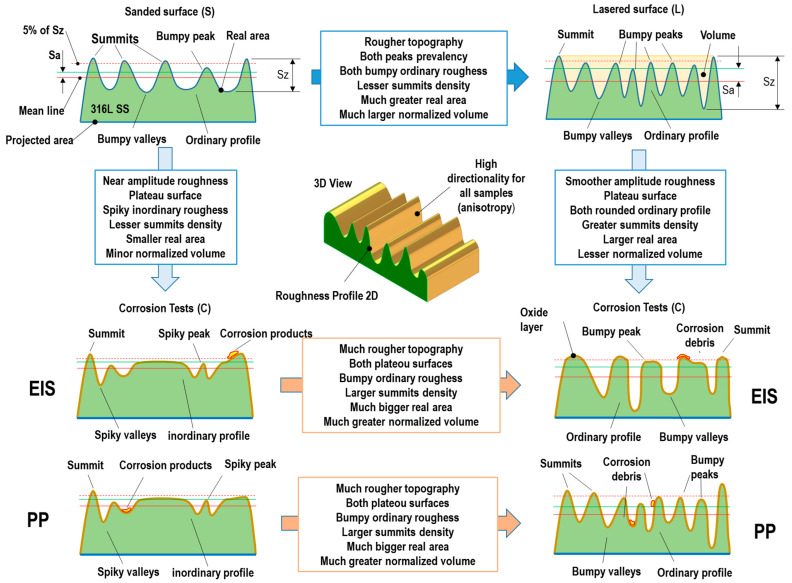
Scheme of the sanded and lasered sample topographies before and after corrosion tests (unscaled sketch).

**Figure 10:**
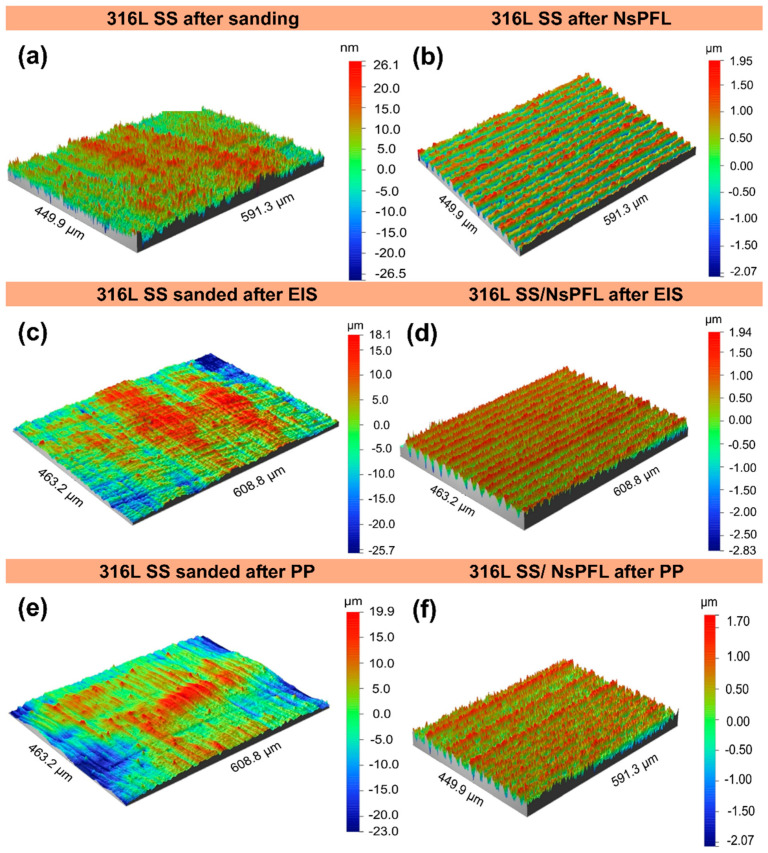
Optical profilometry of (**a**) 316L SS after sanding, (**b**) 316L SS after NsPFL, (**c**) 316L SS sanded after EIS, (**d**) 316L SS/NsPFL after EIS, (**e**) 316L SS sanded after PP, and (**f**) 316L SS/NsPFL after PP.

Corrosion tests practically did not change the initial texture directionality pattern, since the texture aspect ratio (Str) kept values very close to zero, mainly for the laser sample (Str = 0.02 and 0.03), however the sanded sample suffered a tiny alteration towards an isotropic surface for both EIS (Str = 0.29) and PP (Str = 0.37), respectively. Despite Str increasing by about five times this trend cannot be considered significant. Finally, an opposite behaviour was found regarding the surface index (SI) after corrosion tests for sanded and laser samples, regardless of the normalised volume (V). While the real sanded surface reduced from 93.3% to 96.6%, the lasered specimen augmented by 10.65% and 50.94%, respectively, for PP and EIS corrosion tests. In addition, the volume occupied between the highest peak and lowest valley decreased around 15% for the sanded surface and 9% for the laser-texturized samples. In other words, even increasing the real lasered area after corrosion tests, the normalised volume decrement associated with the skewness results means that some displacement of material such as corrosion products was deposited on valleys obscuring electroactive surface areas. Figure 9 displays a detailed schematic drawing based on roughness parameters from Table 1 and subsequent explanations by which it is possible to visualise all 2D configurations of the textures produced before and after corrosion tests. Figure 10 shows the images obtained from optical profilometry on the 316L SS and 316L SS/NsPFL specimens before and after the corrosion tests.

## 4. Conclusions

Considering applications in environments containing chloride ions, the laser-treated 316L SS sample exhibited markedly superior uniform corrosion performance compared to the base material after 48 h of immersion in 0.9 wt% NaCl solution. This improvement is particularly noteworthy considering the widespread utilisation of 316L SS in the fabrication of surgical instruments, where resistance to corrosion is essential for ensuring the longevity and safety of the tools in clinical settings. The enhanced corrosion resistance observed in the laser-treated sample not only underscores its potential for extended use in challenging environments, but also highlights the importance of innovative surface treatment technologies in advancing the reliability of materials used in medical applications. Further comprehensive studies should be conducted to assess the efficacy of NsPFL treatment in surgical devices that may be subjected to diverse aggressive environments, including blood, alcohol, and certain disinfectants such as hydrogen peroxide.

## Figures and Tables

**Table 1:** Roughness parameters used to characterise the specimen surfaces as a function of the corrosion tests (percentile variability ranged from 5.1% to 17.9%).

Specimen	Sa (nm)	St (nm)	Ssk (-)	Sku (-)	Sds (mm^−2^)	Str (-)	SI (%)	V (μm^3^/μm^2^)
Sanded	6.25	52.64	0.03	2.95	4993.95	0.07	0.0030	0.01803
Laser	746.02	4020	0.08	1.74	2065.54	0.03	5.2093	1.47097
Sanded + EIS	6.23	47.82	−0.46	3.34	1403.11	0.29	0.0002	0.01528
Laser + EIS	705.82	4770	−0.45	2.65	3172.60	0.02	7.8628	1.36172
Sanded + PP	6.44	44.48	−0.49	3.15	1286.21	0.37	0.0001	0.01533
Laser + PP	565.24	4040	−0.03	2.31	2518.72	0.02	5.7656	1.30077

## Data Availability

The original contributions presented in this study are included in the article. Further inquiries can be directed to the corresponding author.
